# Postnatal pediatric systemic antibiotic episodes during the first three years of life are not associated with mode of delivery

**DOI:** 10.1371/journal.pone.0229861

**Published:** 2020-03-04

**Authors:** Dominick J. Lemas, Jasmine A. Mack, Jennifer J. Schoch, Nicole Cacho, Elizabeth Plasencia, Alice S. Rhoton-Vlasak, Josef Neu, Lindsay Thompson, Magda Francois, Keval Patel, William R. Hogan, Gloria P. Lipori, Matthew J. Gurka

**Affiliations:** 1 Department of Health Outcomes and Biomedical Informatics, University of Florida College of Medicine, Gainesville, Florida, United States of America; 2 Department of Dermatology, University of Florida College of Medicine, Gainesville, Florida, United States of America; 3 Department of Pediatrics, University of Florida College of Medicine, Gainesville, Florida, United States of America; 4 Department of Obstetrics and Gynecology, University of Florida College of Medicine, Gainesville, Florida, United States of America; 5 University of Florida Health Shands Hospital, Gainesville, Florida, United States of America; University of New South Wales, AUSTRALIA

## Abstract

**Background:**

Delivery by cesarean section (C-section) is associated with adverse short-term and long-term infant outcomes. Given that antibiotics during early life are prescribed for infant outcomes that are more likely among c-section deliveries, we hypothesized that postnatal antibiotic exposure will be greater among c-section infants compared to vaginally delivered infants.

**Objective:**

The aim of this paper was to evaluate if mode of infant delivery was associated with patterns of systemic antibiotic exposure in children during their first three years.

**Methods:**

Pediatric electronic health records from UFHealth, 2011 to 2017 were reviewed. We included singleton, term infants (37–42 weeks gestation) with a birth weight ≥ 2500 grams, with documented mode of delivery and well visits on record. Infants with a neonatal intensive care unit stay were excluded. Both oral and intravenous antibiotics for a 10-day duration were classified as a single episode. The primary outcome was antibiotic episodes in the first three years of life, and a sub-analysis was performed to compare broad-spectrum versus narrow-spectrum antibiotic exposures.

**Results:**

The mean number of antibiotic episodes in 4,024 full-term infants was 0.34 (SD = 0.79) and 24.1% of infants had at least one antibiotic episode. Penicillins were the most prescribed antibiotic in children 0–1 years (66.9%) and cephalosporins were the most common antibiotic prescribed for children 1–3 years (56.2%). We did not detect a meaningful or significant rate ratio (RR) between mode of delivery and overall antibiotic episodes 1.14 (95% CI 0.99, 1.31), broad-spectrum episodes 1.19 (95% CI 0.93, 1.52, or narrow-spectrum episodes 1.14 (95% CI 0.97, 1.34).

**Conclusion:**

Our results do not support the hypothesis that postnatal antibiotic exposure was greater among infants delivered by cesarean section compare to infants delivered vaginally during the first three years of life.

## Introduction

Cesarean section (CS) is the most common surgical procedure in the United States[1] and accounts for approximately 30% of infant deliveries[2]. Delivery by cesarean section has been associated with adverse health outcomes for both infant and mother,[3,4] and CS rates beyond 10–15% may be potentially avoidable[3–5]. Short-term health outcomes for infants delivered via CS include increased respiratory distress[4,6–8], delayed breastfeeding, hypoglycemia[3,9], suppressed immune function[3,10], blood pressure abnormalities[3], altered thermogenic response[3], and increased admissions to the NICU[8,11]. Long-term adverse health outcomes for CS deliveries include increased risk of immune-related conditions such as asthma,[3,4,10,12–14] Type 1 diabetes,[3,12] food allergies,[3,4,10] allergic rhinitis[3,4,10] and celiac disease,[9,15] as well as outcomes related to obesity [3,12,16–21]. The human microbiome has emerged as a potential therapeutic mechanism that links CS with adverse infant health outcomes [22]. One mechanism believed to link CS and later disease is the hygiene hypothesis and the differential exposure to maternal microbiota experienced by infants born via CS versus vaginal delivery [22]. Despite these observations, population-level data has produced conflicting results in implicating mode of delivery as a critical factor contributing to infant health outcomes [16,23]. A potential explanation for this inconsistency in outcomes may be residual confounding by early life exposures such as postnatal antibiotics [24].

Antibiotics are among the most common drug class prescribed to children [25] with the highest incidence rates in children under the age of five [26–30]. Overutilization of antibiotics, especially those that are broad-spectrum, remain a public health concern as they promote greater levels of resistance [31]. Postnatal antibiotic exposure in children has been shown to have a direct influence on the infant microbiome [32] and health outcomes such as obesity [16]. Epidemiological data has demonstrated CS with subsequent antibiotic exposure during the first six months of life was associated with increased risk of obesity among boys at age seven [16]. Notably, antibiotics are frequently prescribed for symptoms (asthma, respiratory, and gastrointestinal infections) that are more likely among cesarean delivered infants[33]. Exposure to antibiotics during the first year of life is associated with subsequent antibiotic exposure throughout the life span[34]. Population-based investigation of antibiotics frequently report the effect size of the exposure as antibiotic episodes (1–4+) associated with a change in a given health outcome [35]. Taken together, we hypothesized that postnatal antibiotic exposure will be greater among c-section infants compared to vaginally delivered infants. To address this hypothesis, we leveraged electronic health records (EHRs) to characterize the prevalence of pediatric antibiotic presecriptions during the first three years of life and tested for significant associations between mode of delivery and antibiotic episodes. EHRs are a low-cost, large-scale data source with relatively low error rates [36], especially when compared to manual data extractions, that can be utilized to track population trends in pediatric antibiotic utilization[35].

## Methods

### Study design

De-identified linked maternal-infant electronic health records (EHRs) were collected from UFHealth Shands Hospital and UFHealth Jacksonville between June 1, 2011 and April 30, 2017. Briefly, an honest broker within UF Health Integrated Data Repository (IDR) used fully identified data to perform the linkage, then removed identifiers from the linked data set before releasing the de-identified data to the research team. Demographic information such as insurance type at delivery, age, race, and ethnicity were extracted from the maternal chart and infant demographics including sex, race and ethnicity were extracted from the infant chart. Notably, all infants delivered with the UF Health system are linked to maternal records as a standard of care. The inclusion criteria for maternal-infant pairs included in the analysis is presented in S1 Fig. We included term singleton infants (37–42 weeks gestational age) with a birth weight ≥ 2500 grams, at least one documented well visit during the first 36 months of life, and mode of delivery classified as either “low transverse cesarean section” or “spontaneous vaginal delivery”. To account for the possibility of confounding from premature infants requiring antibiotics, we excluded infants that were premature (<37 weeks gestational age), low birth weight (<2500 grams), or admitted to the neonatal intensive care unit for any reason. We included infants with sequential measurements, usually occurring in the setting of a well visit, during the exposure period in an effort to define a conservative cohort of children with an established connection to UFHealth care services that likely increases the probability of capturing complete antibiotic prescribing data. Taken together, our analysis includes children within the sample that have variable follow-up times within the 36-month time period. This study was approved by the institutional review board at the University of Florida.

### Primary outcome

The primary outcome of interest was systemic antibiotic episodes in the first 36 months of life. We extracted antibiotic prescription information from all clinical encounters, including inpatient, emergency or ambulatory visits at ≤36 months of age. All antibiotics were categorized according to the Anatomic Theraputic Chemical (ATC) index, a hierarchical classification system for the purposes of drug statistics that was developed by the World Health Organization[37]. Systemic antibiotics were defined as all drugs within the ATC-group J01 (antibiotics for systemic use). An antibiotic episode was defined as any systemic antibiotic that was administered or prescribed more than 10 days from any prior or subsequent antibiotic prescription, from birth to the last wellness visit on record. Cumulative antibiotic episodes were analyzed as a count variable through the last antibiotic prescription within the first three years. We also separately examined narrow-spectrum and broad-spectrum antibiotic episodes for each child. Narrow spectrum antibiotics were defined as those that act on one major group of bacteria. Broad spectrum antibiotics were defined as those that act on two or more major bacterial groups (S1 Table). Within the analytical sample, the antibiotic episodes ranged from 0 to 12 occurrences (narrow-spectrum: 0–11, broad-spectrum: 0–6). Due to the nature of multiple prescriptions and exposure windows, children may have simultaneous exposure to narrow- and broad-spectrum antibiotics, thus these drug categories are not mutually exclusive. For example, if a child receives narrow and broad-spectrum antibiotics within the same 10-day window, the occurrence will count as one antibiotic episode. However, it will also count as one episode each under the narrow and broad-spectrum antibiotic categories. We excluded topical antibiotics as there is no evidence linking topical antibiotics to the gut microbiome or infant outcomes such as weight gain.

### Statistical analysis

We evaluated the association between mode of delivery and the rate of antibiotic episodes over the first three years via negative binomial regression, which accounted for differential follow-up time per child (S1 Fig). We also modeled separately by antibiotic: narrow-spectrum and broad-spectrum classes. To assess for possible differences over time, cumulative antibiotic counts over the first three years were modeled and compared between the two delivery groups via recurrent events analysis within a proportional hazards regression framework. Unadjusted rate ratios and hazard ratios (and 95% confidence intervals (CI)) comparing the delivery groups were estimated and reported for the two model types, respectively. We also adjusted for infant gender and covariates found to be different between the two delivery groups; these adjusted rate and hazard ratios (and 95% CI’s) were also reported. We assessed for collinearity of these covariates with variance inflation factors (VIF) before adding covariates to the adjusted model, where VIF>5 signifying collinearity. Chi-squared analyses and two-sample t-tests were used to compare demographic characteristics by delivery group. Analyses were performed with a significance level of *α =* 05 using SAS software version 9.4 (SAS Institute, Cary, NC).

## Results

### Study cohort

We obtained data on 16,684 infants born between June 1, 2011 and April 30, 2017. Of these, 4,024 (24.1%) children with at least one well visit met inclusion criteria (S1 Fig). Mother and child pairs were categorized by mode of delivery, where 1,211 (30.1%) infants were delivered by cesarean section and 2,813 (69.9%) were delivered vaginally. Infants in either the cesarean section or vaginal delivery group were equally distributed by gender and there was no statistically significant difference in race by group, as shown in Table 1. Infants in the vaginal delivery group were gestationally older (39.5 weeks, SD = 1.1 vs 39.3 weeks, SD = 1.1, *p* < .0001) and had smaller birthweight (3315 grams, SD = 411 vs 3375 grams, SD = 460, *p* = 0.0001) compared to children in the cesarean section group. Mothers who delivered either by cesarean section or vaginally had similar race/ethnicity and health insurance profiles. Those who gave birth by cesarean section were older (28.4 years, SD = 5.7 vs 27.4 years, SD = 5.7, *p* < .0001).

**Table 1:** Characteristics of the infant and maternal population by mode of delivery^1^^,^^2^.

	Total n = 4,024	Cesarean Section n = 1,211	Vaginal Delivery n = 2,813	*p*-value
**Infant Characteristics**				
Gender				
*Female*	49.3	47.8	49.9	0.2297
*Male*	50.7	52.2	50.1	
Race/Ethnicity				
*Non-Hispanic White*	41.3	42.1	40.9	0.3139
*Non-Hispanic Black*	33.4	34.5	32.9	
*Non-Hispanic Other*	16.9	15.6	17.5	
*Hispanic*	8.4	7.8	8.7	
Gestational Age (weeks), M (SD)	39.4 (1.1)	39.3 (1.1)	39.5 (1.1)	**< .0001**
Baby Birth Weight (grams), M (SD)	3333 (427)	3375 (460)	3315 (411)	**0.0001**
**Maternal Characteristics**				
Maternal Age, M(SD)	27.7 (5.7)	28.4 (5.7)	27.4 (5.7)	**< .0001**
Race/Ethnicity				
*Non-Hispanic White*	45.2	44.7	45.5	0.6937
*Non-Hispanic Black*	32.8	34.1	32.2	
*Non-Hispanic Other*	13.9	13.5	14.2	
*Hispanic*	8.0	7.8	8.1	
Insurance				
*Private*	31.8	32.0	31.7	0.4714
*Public (Medicare/Medicaid/CMS)*	53.9	54.8	53.5	
*Managed Care/Other*	14.3	13.3	14.8	

^1^Singleton infants included in the analysis possessed a gestational age greater than or equal to 37 weeks and less than or equal to 42 weeks, birth weight greater than 2500 grams, no time in the NICU, well visits on record and systemic antibiotic data only within the first three years of life and have demographics, a date of birth, and mode of delivery on record.^1^Percentage unless otherwise noted.

^2^Chi-squared analyses were used to compute p-values for categorical covariates and two-sample t-test for continuous covariates.

**Fig 1:**
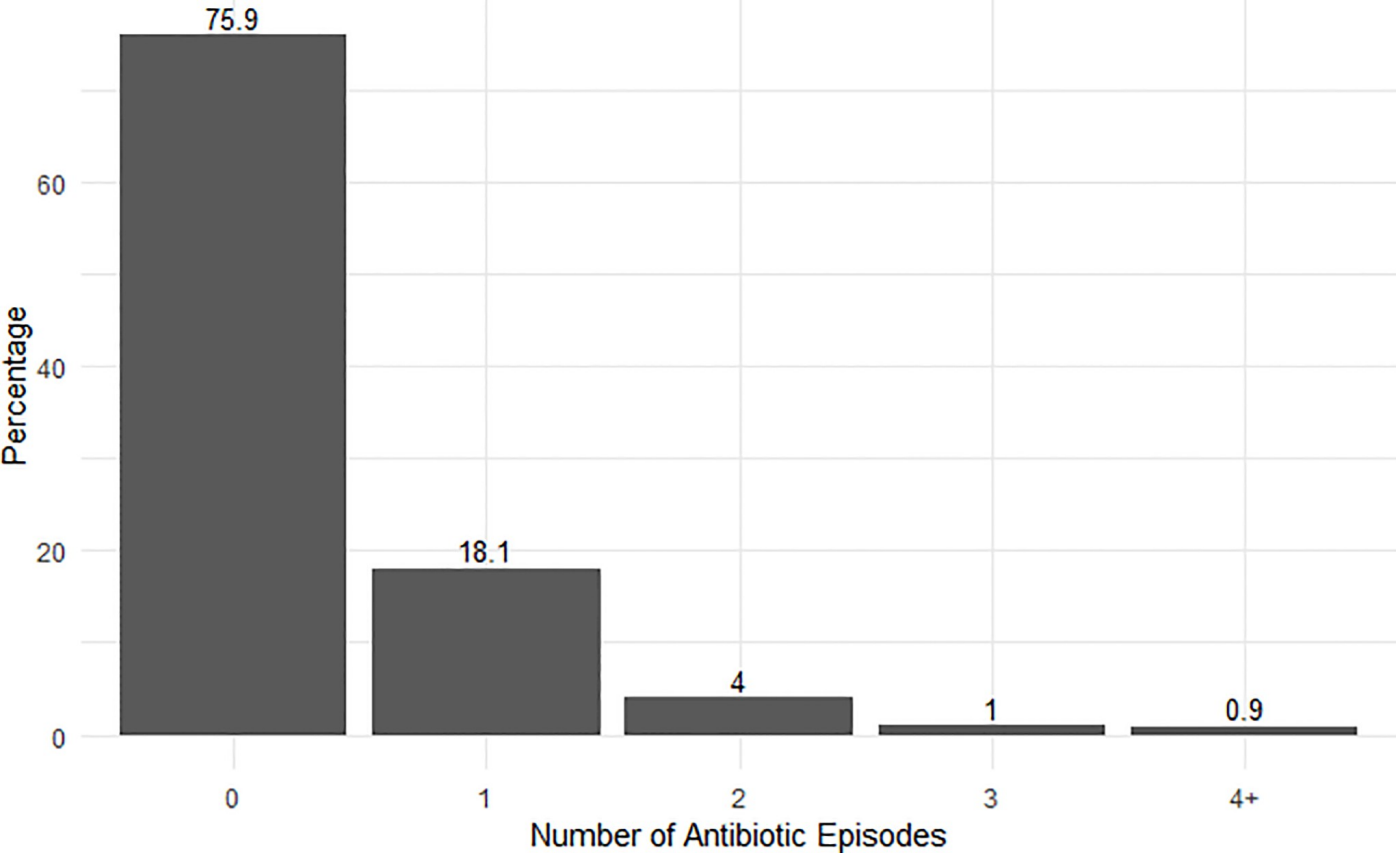
Postnatal pediatric antibiotic episodes per child. Antibiotic episodes were defined as any antibiotic that was administered or prescribed more than 10 days from any prior or subsequent antibiotic prescription, from birth to the last wellness visit on record. Cumulative antibiotic episodes for each child were analyzed as a count variable and shown here as the percentage of infants that were prescribed 0 to 4 or more antibiotic episodes during the first three years.

**Fig 2:**
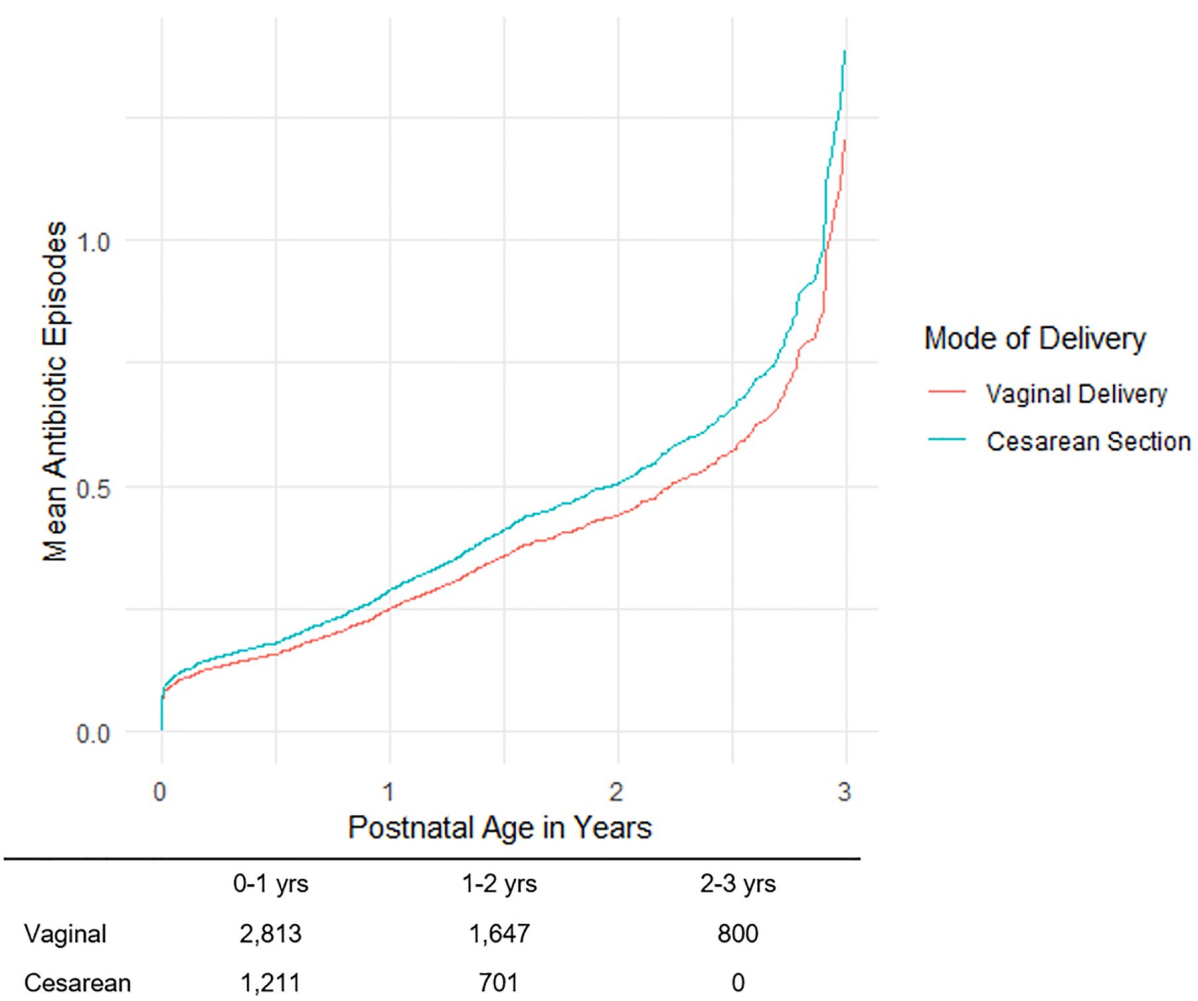
Recurrent postnatal antibiotic episodes by mode of delivery. After adjusting for covariates, there was no statistically significant difference between the number of total antibiotics episodes (p = 0.07). In this line chart, the mean antibiotic episode was estimated over the first three years of life by mode of delivery. Blue represents those infants delivered via cesarean section and the orange represents vaginally delivered infants. Counts of infants according to mode of delivery and postnatal age are listed below the line chart.

Of 1,375 antibiotic prescriptions in 970 infants, penicillins, which includes Ampicillin, (66.9%) were the most prescribed antibiotic class in children 0–1 years (Table 2). We also found that cephalosporins were the most common antibiotic class for children 1–2 years (56.2%) and 2–3 years (56.2%). The mean number of antibiotic episodes over 3 years in all children was 0.34 (SD = 0.79), and 24.1% of infants had at least one antibiotic episode during this period. For those children that had at least one episode, the mean number of antibiotic episodes was 1.42 (SD = 1.02). Fig 1 shows that 75.9% of infants in this study did not receive any antibiotics and 18.1% of children received only a single course of antibiotics. S2 Table shows that 18.8% of children received at least one episode of narrow spectrum antibiotics and 8.1% of children received at least one broad spectrum antibiotic episode during the first 3 years of life. As shown in Table 3, babies born by cesarean section had 0.37 episodes after three years (95% CI: 0.32, 0.43) while those born vaginally had 0.33 episodes (95% CI: 0.30, 0.35). Fig 2 shows the recurrent postnatal antibiotic episodes by mode of delivery. After adjusting for covariates, there was no statistically significant difference between the number of total antibiotics episodes (Table 3, Rate Ratio (RR) = 1.14, 95% CI: (0.99, 1.31), p = 0.0758; S3 Table, RR = 1.15 (0.98, 1.34), p = 0.0796), including narrow-spectrum (Table 4, RR = 1.14, 95% CI: (0.97, 1.34), p = 0.1031) and broad-spectrum episodes (Table 5, RR = 1.19, 95% CI: (0.93, 1.52), p = 0.1654). No collinearity was observed in any of the statistical models (VIF < 2).

**Table 2:** Antibiotic prescriptions by ATC code among infants during first 3 years of life^1^.

ATC-code	ATC Name	Age Group 0<1 years	Age Group 1–2 years	Age Group 2–3 years	All
J01A	Tetracyclines	0 (0.0%)	1 (0.3%)	0 (0)	1 (0.1%)
J01C	Beta-lactam antibacterials, penicillins	588 (66.9%)	136 (39.0%)	59 (40.4%)	783 (57.0%)
J01D	Other beta-lactam antibacterials	260 (29.6%)	196 (56.2%)	82 (56.2%)	538 (39.1%)
J01E	Sulfonamides and trimethoprim	3 (0.3%)	2 (0.6%)	1 (0.7%)	6 (0.4%)
J01F	Macrolides, lincosamid and streptogramins	9 (1.0%)	6 (1.7%)	1 (0.7%)	16 (1.2%)
J01X	Other antibacterials	19 (2.2%)	8 (2.3%)	3 (2.1%)	30 (2.2%)
J04A	Drugs for treatment of tuberculosis	1 (0.1%)	0 (0.0%)	0 (0.0%)	1 (0.1%)
	Total	880	349	146	1,375

^1^Antibiotic count and (%).

**Table 3:** Total Antibiotic episodes by mode of delivery.

Mode of Delivery	N	Total Antibiotics Episode (95% CI)	*Negative Binomial Rate Ratio (95% CI)*	
			Unadjusted	Adjusted^1^
**Cesarean Section**	1,211	0.37 (0.32, 0.43)	1.13 (0.98, 1.30)	1.14 (0.99, 1.31)
			*p = 0*.*1004*	*p = 0*.*0758*
**Vaginal Delivery**	**2,813**	0.33 (0.30, 0.35)	Ref	Ref

^1^Adjusted for infant race, sex, birth weight, gestational age and maternal age.

**Table 4:** Narrow spectrum antibiotics by mode of delivery.

Mode of Delivery	N	Narrow Spectrum Antibiotics Episode (95% CI)	*Negative Binomial Rate Ratio (95% CI)*	
			Unadjusted	Adjusted^1^
**Cesarean Section**	1,211	0.27 (0.23, 0.31)	1.13 (0.96, 1.32)	1.14 (0.97, 1.34)
			*p = 0*.*1373*	*p = 0*.*1031*
**Vaginal Delivery**	**2,813**	0.23 (0.21, 0.25)	Ref	Ref

^1^Adjusted for infant race, sex, birth weight, gestational age and maternal age.

**Table 5:** Broad spectrum antibiotics by mode of delivery.

Mode of Delivery	N	Broad Spectrum Antibiotics Episode (95% CI)	*Negative Binomial Rate Ratio (95% CI)*	
			Unadjusted	Adjusted^1^
**Cesarean Section**	1,211	0.13 (0.10, 0.15)	1.20 (0.94, 1.53)	1.19 (0.93, 1.52)
			*p = 0*.*1425*	*p = 0*.*1654*
**Vaginal Delivery**	**2,813**	0.10 (0.08, 0.12)	Ref	Ref

^1^Adjusted for infant race, sex, birth weight, gestational age and maternal age.

## Discussion

We evaluated the association between mode of delivery and pediatric postnatal antibiotic exposure in the first three years among term infants using linked mom-baby EHRs. Our analysis revealed that nearly 1 in 4 infants in our study had at least one postnatal antibiotic episode within the first three years of life. Penicillins (J01C, 66.9%) were the most prescribed antibiotic in children 0–1 years and cephalosporins were the most common antibiotic for children 1–3 years (J01D, 56.2% for 1–2 years and 56.2% for 2–3 years). The primary finding of this study demonstrates that there was no statistical difference in the rate of prescribed antibiotics in children delivered via CS compared to children delivered vaginally in the first three years of life. Although we adjusted for potential confounding factors, the possibility that residual confounding exists within our results is plausible. Nevertheless, the precision of the effect estimates and confidence intervals from our analysis indicates that even if the association between mode of delivery and postnatal pediatric antibiotics at age three were significant, it would not represent a clinically meaningful association, as the 95% CI’s did not contain values that one would consider to represent clinically meaningful differences between the modes of delivery [38]. Taken together, our primary results do not support the hypothesis that otherwise healthy full-term infants delivered via cesarean section are more likely to be prescribed antibiotics than vaginally delivered infants.

The prevalence of pediatric antibiotic episodes in our study are generally consistent with population-based estimates. Holstiege et al 2014, demonstrated that among five European countries, the rates of prescriptions were highest in young children (≤4 years) in all countries, predominantly due to high use of broad spectrum penicillins [39]. In a cohort of US children (1–17 years), Sarpong et al. reported more than a quarter (27.3%) of children used antibiotics with 12.8% using broad2010030spectrum antibiotics and 18.5 percent using narrow‐spectrum antibiotics [27]. Notably, our analysis in children 0–3 years of age revealed that nearly 1 in 5 (18.8%) children received at least one episode of narrow spectrum antibiotics and 8.1% of children received at least one broad spectrum antibiotic episode during the first 3 years of life. Our results also revealed that more than 65% of pediatric antibiotics were prescribed to children less than one year of age and that antibiotics classified as beta-lactams (J01C and J01D) accounted for more than 95% of all postnatal prescriptions during the first three years of life. It is worth noting that cephalosporins (J01D) in our analysis were the most common antibiotic for children 1–3 years of age. Consistent with our results, Resi et al. reported that cephalosporins were the most commonly prescribed antibiotic among children under the age of 7 years [40]. Collectively, our results suppport the importance of electronic health records as an accurate and accessible information source to track population trends in antimicrobial stewardship.

The impact of antibiotic exposure and child-maternal health outcomes has largely focused on intrapartum antibiotic prophylaxis (IAP) to reduce neonatal early-onset sepsis (EOS) [41]. As a standard of care, most women receive IAP either for Group B Streptococcus (GBS) colonization during labor[42], or for unknown GBS status during preterm labor. IAP is administered as a form of prophylaxis for infection prior to cesarean delivery[43,44] and for preterm premature rupture of membranes (PPROM) prior or during labor and delivery [45]. Our analysis did not formally address IAP or perinatal antibiotics; however we were able to adjust for early-onset sepsis (EOS) risk factors and excluded children with birth weight < 2500 grams and admission to the neonatal intensive care unit (NICU). Additionally, our analysis likely included infants that were treated with empiric antibiotics for presumed sepsis during the first 48 hours, receiving antibiotics without NICU admission. Previous work has demonstrated ~6% of term infants treated with empiric antibiotics for presumed sepsis were exposed to ampicillin and/or gentamicin immediately after birth [46,47]. Additional limitations of this study included limited verification of patient antibiotic compliance and bias associated with lack of follow-up/missing data. EHR data. There is also potential for misclassification of pediatric antibiotic prescriptions and the possibility that children are receiving pediatric care (and antibiotics) outside the UFHealth system. We would anticipate that any misclassification is nondifferential with respect to delivery type, which admittedly would bias comparisons in rates between the delivery groups towards the null. There is also potential for antibiotic episodes calculated from inpatient and outpatient prescriptions to include antibiotics that were prescribed but may not have been taken. As described previously[35,48], we computed antibiotic episodes using a 10-day window and it is possible a different window of time in between antibiotic prescriptions may result in differences by mode of delivery. Notably, we found that two percent of the infants were administered narrow-spectrum and broad-spectrum within the same 10-day window, thus the occurrences of narrow and broad episodes are not mutually exclusive.

Cesarean sections delivery and pediatric antibiotic exposure have each independently been associated with changes in pediatric outcomes such as obesity [49] and asthma [50]. Despite these observations, there is limited population-based data that describes how mode of delivery informs the patterning of postnatal pediatric antibiotic prescriptions. In a large Canadian cohort (n = 251,817) focused on antibiotics and the risk of pediatric asthma, infants that were delivered by CS accounted for 24% of births and 43% of these children were prescribed at least one antibiotic in their first year of life [51]. An analysis of antibiotics and pediatric obesity in 28,354 Danish mother-child dyads revealed that exposure to antibiotics during the first six months of life was the same between children delivered vaginally and children delivered by CS [16]. Our results extend these observations by reporting mode of delivery, in healthy term infants, was not associated with overall antibiotic episodes, narrow-spectrum or broad-spectrum episodes during the first three years of life.

Our study has several strengths. It is the largest population-based study to examine this question with more than 4,000 children followed for up to three years. We also adjusted for potential confounding factors that have not been previously examined including method of delivery and the presence of birth complications. In addition, our birth cohort was large enough to adequately examine multiple courses of antibiotics and the impact of mode of delivery on broad and narrow-spectrum antibiotics. This study indicates that mode of delivery in healthy full term infants should not be influenced by concern for future antibiotic use of infant. Although providers should continue to strive for lower cesarean section rates in the United States [5]; our results demonstrate that pediatric systemic antibiotic prescriptions are not significantly different between infants born by cesarean section and those born vaginally during the first three years of life.

## Supporting information

S1 Fig Flow chart of pediatric participants. (DOCX)

S1 Table Classification of narrow spectrum and broad spectrum antibiotics. (DOCX)

S2 Table Pediatric antibiotic episodes per child during the first three years of life. (DOCX)

S3 Table Cox proportional hazard model of recurrent events. (DOCX)
