# Supplementary material for: Postnatal pediatric systemic antibiotic episodes during the first three years of life are not associated with mode of delivery
Source: PLoS One. 2020 Mar 4;15(3):e0229861. doi: 10.1371/journal.pone.0229861 (PMC7055886; doi:10.1371/journal.pone.0229861)
Supplement: S1 Fig — (DOCX) [file pone.0229861.s001.docx]

**
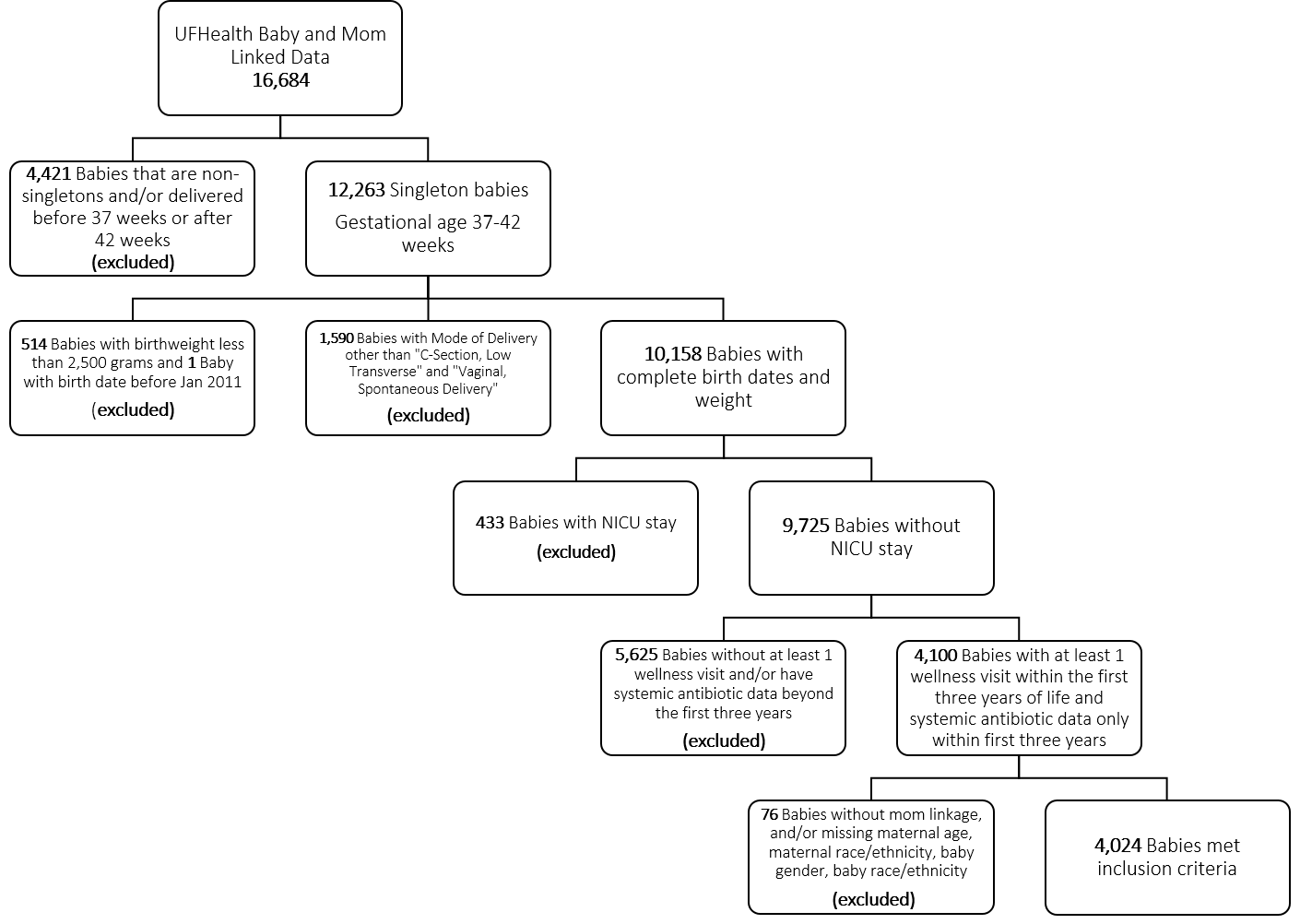
**

**S1 Fig.** Flow chart of pediatric participants included in the analysis from UFHealth electronic health records between June 1, 2011 and April 30, 2017.
