# Supplementary material for: Postnatal pediatric systemic antibiotic episodes during the first three years of life are not associated with mode of delivery
Source: PLoS One. 2020 Mar 4;15(3):e0229861. doi: 10.1371/journal.pone.0229861 (PMC7055886; doi:10.1371/journal.pone.0229861)
Supplement: S1 Table — (DOCX) [file pone.0229861.s002.docx]

**S1 Table. Classification of narrow spectrum and broad spectrum antibiotics.**

| **Narrow** | |  | **Broad** | |
| --- | --- | --- | --- | --- |
| ***Penicillin*** | amoxicillin, penicillin g benzathine, penicillin g sodium, penicillin g procaine, oxacillin |  | ***Penicillin*** | amoxicillin-clavulanate, ampicillin, ampicillin-sulbactam, penicillin v potassium |
| ***Glycopeptide*** | vancomycin |  | ***Cephalosporins*** |  |
|  |  |  | ***First-generation*** | cefadroxil, cefalexin |
|  |  |  | ***Second-generation*** | cefaclor, cefoxitin, cefprozil, cefuroxime |
|  |  |  | ***Third-generation*** | cefixime, cefpodoxime, ceftriaxone |
|  |  |  | ***Macrolides*** | clarithromycin, erythromycin, erythromycin-sulfisoxazole |
|  |  |  | ***Tetracyclines*** | doxycycline |
|  |  |  | ***Lincosamides*** | clindamycin |
|  |  |  | ***Sulfonamides/ trimethoprim*** | sulfamethoxazole-trimethoprim |
|  |  |  | ***Nitrofurans*** | nitrofurantoin |
|  |  |  | ***Polypeptides*** | polymyxin b trimethoprim |
|  |  |  | ***Quinolones*** |  |
|  |  |  | ***First-generation*** | ciprofloxacin |
|  |  |  | ***Rifamycins*** | rifampin |
|  |  |  | ***Miscellaneous/Combination*** | aztreonam, bacitracin-neomycin-polymyxin, neomycin-polymyxin, metronidazole |
