# Supplementary material for: Postnatal pediatric systemic antibiotic episodes during the first three years of life are not associated with mode of delivery
Source: PLoS One. 2020 Mar 4;15(3):e0229861. doi: 10.1371/journal.pone.0229861 (PMC7055886; doi:10.1371/journal.pone.0229861)
Supplement: S2 Table — (DOCX) [file pone.0229861.s003.docx]

**S2 Table. Pediatric antibiotic episodes per child during the first three years of life (n=4,024).**

| Antibiotic Episode | Total | | Narrow | | | Broad | | | |
| --- | --- | --- | --- | --- | --- | --- | --- | --- | --- |
|  | Number of Children | Percent of all Children (%) | Number of Children | | Percent of all Children (%) | Number of Children | | Percent of all Children (%) |  |
| 0 | 3054 | 75.9 | 3263 | 81.1 | | 3697 | 91.9 | | |
| 1 | 730 | 18.1 | 633 | 15.7 | | 268 | 6.7 | | |
| 2 | 161 | 4.0 | 90 | 2.2 | | 41 | 1.0 | | |
| 3 | 42 | 1.0 | 26 | 0.6 | | 11 | 0.3 | | |
| 4+ | 19 | 0.5 | 12 | 0.3 | | 6 | 0.1 | | |
