# Supplementary material for: Postnatal pediatric systemic antibiotic episodes during the first three years of life are not associated with mode of delivery
Source: PLoS One. 2020 Mar 4;15(3):e0229861. doi: 10.1371/journal.pone.0229861 (PMC7055886; doi:10.1371/journal.pone.0229861)
Supplement: S3 Table — (DOCX) [file pone.0229861.s004.docx]

**S3 Table. Cox proportional hazard model of recurrent events**

| **Mode of Delivery** | **N** | **Cox PH Model of Recurrent Events Hazard Ratio (95% CI)** | |
| --- | --- | --- | --- |
|  |  | Unadjusted | Adjusted^1^ |
| **Cesarean Section** | 1,211 | 1.14 (0.98, 1.33) | 1.15 (0.98, 1.34) |
|  |  | *p=0.1020* | *p=0.0796* |
| **Vaginal Delivery** | **2,813** | Ref | Ref |
